# Beyond amyloid: nanobody-mediated neuroinflammatory therapy for Alzheimer’s disease

**DOI:** 10.1186/s40035-025-00513-5

**Published:** 2025-10-13

**Authors:** Soukaina Amniouel, Jessica Suh, Wei Zheng, Qi Zhang

**Affiliations:** Therapeutic Development Branch, National Center for Advancing Translational Sciences, National Institutes of Health, Bethesda, MD 20892 USA

**Keywords:** Alzheimer’s disease, Single-domain antibody, Anti-neuroinflammation therapy, Antibody-drug conjugate, sdAb, Neurodegenerative disease

## Abstract

Alzheimer’s disease (AD) is one of the most common and devastating neurodegenerative diseases, characterized by accumulation of amyloid-beta (Aβ) plaques, neurofibrillary tangles of tau protein, and persistence of neuroinflammation, leading to progressive cognitive decline, loss of independence, emotional and financial strain on families, and significant societal costs. Current anti-amyloid treatments are partly successful in removing Aβ amyloid, but often lead to increased inflammation. This leads to limited therapeutic efficacy and causes side effects such as amyloid-related imaging abnormalities. In addition, they do not address neuroinflammation in AD patients. In this review, we discuss a new therapeutic strategy that combines single-domain antibodies (sdAbs, nanobodies) against Aβ fibrils and anti-inflammatory drugs and applies them to the regions of neuroinflammation associated with the plaques in AD patients. This strategy aims to control the function of activated microglia and astrocytes, thereby avoiding unnecessary immunosuppression. We also discuss the unique features of sdAbs, including small size, good tissue penetration, and lack of Fc-mediated immune reactions, as well as relevant payloads (i.e., small molecules, biologics, and nanoparticles) and delivery systems. This immunomodulatory therapy targets the plaques specifically and therefore represents a promising opportunity to improve amyloid clearance and target the inflammatory components of AD, potentially improving the therapeutic efficacy of the disease.

## Introduction

Alzheimer’s disease (AD) is a multifactorial neurodegenerative disorder featured by amyloid-beta (Aβ) plaques and intraneural neurofibrillary tangles composed of tau proteins [1]. Chronic neuroinflammation has also been identified as an important component of AD pathogenesis. In AD, the interactions of peripheral immune cells (e.g., neutrophils, leukocytes, monocytes, and T-cells) with central nervous system (CNS)-resident immune cells such as activated microglia and reactive astrocytes significantly contribute to neurodegeneration through exacerbating blood–brain-barrier (BBB) dysfunction and neuroinflammation [2]. Neutrophils infiltrate a compromised BBB in response to Aβ accumulation and inflammatory signals [3]. The migration across endothelial cells releases proteolytic enzymes and reactive oxygen species (ROS), which collectively degrade tight junctions and enhance BBB permeability [4]. Furthermore, neutrophils release pro-inflammatory cytokines (e.g., tumor necrosis factor-α [TNF-α], interleukin [IL]-1β, IL-12, IL-6) and neutrophil extracellular traps, stimulating microglial activation and astrocyte reactivity, increasing their pro-inflammatory phenotype [3]. Leukocytes similarly disrupt the BBB through adhesion and transmigration, secreting inflammatory mediators such as IL-17 and IFN-γ (interferon gamma) that amplify microglial and astrocytic inflammation [4]. Monocytes recruited to the brain via chemokine signaling (specifically CCL2 [chemokine (C–C motif) ligand 2]) degrade endothelial barriers, differentiate into macrophages, and produce cytokines, increasing inflammation and inducing reactive astrocytic phenotypes [5]. T cells also significantly impair BBB through adhesion molecule-mediated interactions, releasing cytokines upon entry, which promote a sustained inflammatory response in microglia and astrocytes [6]. Activated microglia in response to these signals adopt a pro-inflammatory phenotype, producing cytokines and ROS, thereby perpetuating neuronal damage and plaque accumulation [7]. Reactive astrocytes lose their neuroprotective capabilities, further contributing to neuronal death [7]. This complex interplay of immune cell activity highlights neuroinflammation as a critical therapeutic target for AD [4, 8, 9].

Current treatments for AD are primarily directed at Aβ or tau pathology with limited success. For instance, anti-amyloid monoclonal antibodies (mAbs) such as aducanumab, lecanemab, and donanemab can decrease amyloid burden and slightly improve cognition [10]. However, such treatments do not directly act on the inflammatory microenvironment that is accompanied by amyloid-related imaging abnormalities (ARIA) [11, 12]. On the other hand, nonsteroidal anti-inflammatory drugs (NSAIDs) and some other broad-spectrum anti-inflammatory agents have failed to demonstrate any consistent beneficial efficacy in clinical trials [13]. This may be partially due to suboptimal administration timing and other interlinked factors, such as the stage of disease progression, the complexities of crossing the BBB, dosing limitations, and off-target effects [14–16]. Therefore, new-generation therapies that can mitigate neuroinflammation along with activity against amyloid pathology are needed to improve therapeutics efficacy and long-term outcomes.

**Fig. 1:**
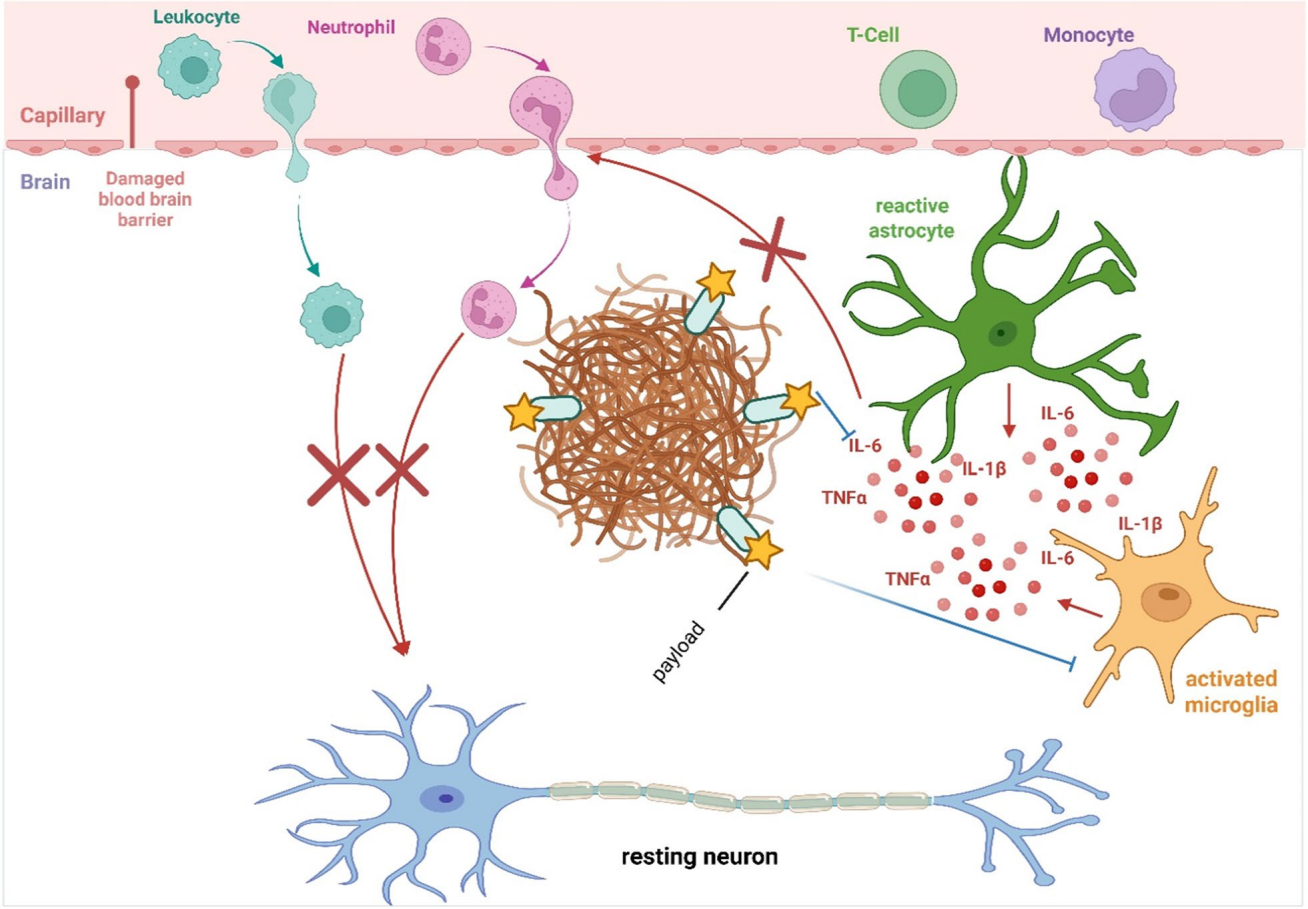
Schematic representation of nanobody-mediated delivery of anti-inflammatory payloads targeting amyloid-β (Aβ) plaques in Alzheimer’s disease (AD). At the center, a dense, irregularly shaped Aβ plaque illustrates the pathological hallmark of AD. Multiple single-domain antibodies (sdAbs) are shown bound to the plaque surface, represented by compact oval shapes to emphasize their small size relative to conventional antibodies. Each sdAb is conjugated to an anti-inflammatory payload, visualized as stars to indicate different therapeutic modalities such as small molecules, cytokines, RNA-based agents, or nanoparticles. Surrounding the plaque, activated microglia and astrocytes reflect the neuroinflammatory environment. Arrows emanating from these glial cells represent the secretion of pro-inflammatory cytokines (e.g., IL-1β, TNF-α). Inhibitory arrows from the sdAb-conjugated payloads point toward either the cytokines or the glial cells, symbolizing the suppression of inflammatory signaling

In this review, we summarize existing data on anti-amyloid and anti-inflammation therapies in the therapeutics development for AD. We outline current approaches in anti-amyloid therapy, including efforts to reduce amyloid burden or interfere with its aggregation and toxicity. We further describe the application of high-specificity anti-Aβ fibril single-domain antibodies (sdAbs), also widely known as nanobodies, as an anti-amyloid therapy and as a targeted delivery system for anti-inflammatory treatment (Fig. 1). By precisely directing treatment to brain regions enriched with Aβ deposits, sites typically characterized by robust activation of microglia and astrocytes, this approach aims to potently inhibit local inflammatory responses while substantially minimizing systemic side effects. In addition, by inhibiting glia-derived pro-inflammatory cytokines, sdAb-payloads may also reduce peripheral immune cell infiltration into brain parenchyma, thereby limiting secondary inflammatory damage to neurons.

## Neuroinflammation in AD: a double-edged sword

AD demonstrates substantial variability in genetic, pathological, and clinical progression aspects. Familial AD often involves mutations in *APP*, *PSEN1*, and *PSEN2*, whereas sporadic AD is genetically complex, influenced by the *APOE* ε4 allele among many other susceptibility genes [17, 18]. Pathologically, AD patients show variability in the distribution and abundance of hallmark features such as Aβ plaques, tau neurofibrillary tangles, and neuroinflammation markers, which influence disease presentation and progression rates [19, 20].

Despite this variability, neuroinflammation is increasingly considered to be a central and consistent feature of AD pathology, having both aggravating and, in the initial stages, mitigating roles [21]. Microglia and astrocytes, the primary immune cells in the brain, demonstrate complex, stage-dependent roles in AD. In the healthy brain, microglia and astrocytes normally serve protective functions, clearing debris and secreting trophic factors to support neuronal homeostasis [22, 23]. However, with the progression of AD, activated microglia are found to aggregate and surround Aβ amyloid plaques, resulting in a proinflammatory phenotype characterized by secretion of cytokines IL-1β, IL-6 and TNF-α [2, 24, 25]. When attempting to remove the Aβ amyloid fibrils, these plaque-associated microglia often have defective phagocytosis [26] and release substances such as ROS [27, 28] and proteases [29] that are neurotoxic and affect neurons and their synapses [24]. During this process, the cellular state can shift to a disease-associated microglia (DAM) phenotype, characterized by altered transcriptional profiles, enhanced phagocytic activity, and secretion of pro-inflammatory cytokines [30, 31]. DAMs downregulate homeostatic genes and upregulate pathways for phagocytosis and lipid metabolism. This activation occurs in a two-step process: an initial triggering receptor on myeloid cells 2 (TREM2)-independent phase followed by a TREM2-dependent phase [30]. DAMs were found clustered around amyloid plaques with ingested Aβ fibrils, suggesting that they restrict plaque growth and neurodegeneration [30]. Interestingly, these findings demonstrate microglial heterogeneity. At early stages of the disease, microglia can adopt a DAM state that encapsulates plaques, reduce damage, whereas other states drive inflammation. Thus, microglia can have a dual nature, capable of restraining pathology or exacerbating it. For example, depleting microglia in AD mice has complex effects; if done early, amyloid plaques fail to form properly, but chronic microglial removal in later stages does not reduce plaque load and can worsen neuritic damage [32]. Consistently, an editorial summary describes microglia as having “two-faces”, an early TREM2-driven response that is protective, and a later pro-inflammatory response marked by complement C3 that induces neurotoxic astrocytes [33]. In patients, in-vivo imaging further highlights the pathogenic role of microglia. Positron emission tomography (PET) studies show that regions of high microglial activation (using TPO or TREM2 PET tracers) are the same regions where tau tangles are spreading [34]. For example, one work reported that microglial activation strongly correlates with tau propagation across Braak stages and may drive the spread of tau in the presence of Aβ [35]. Such evidence positions microglia as a central orchestrator of neuroinflammation and a modulator of AD progression.

**Fig. 2:**
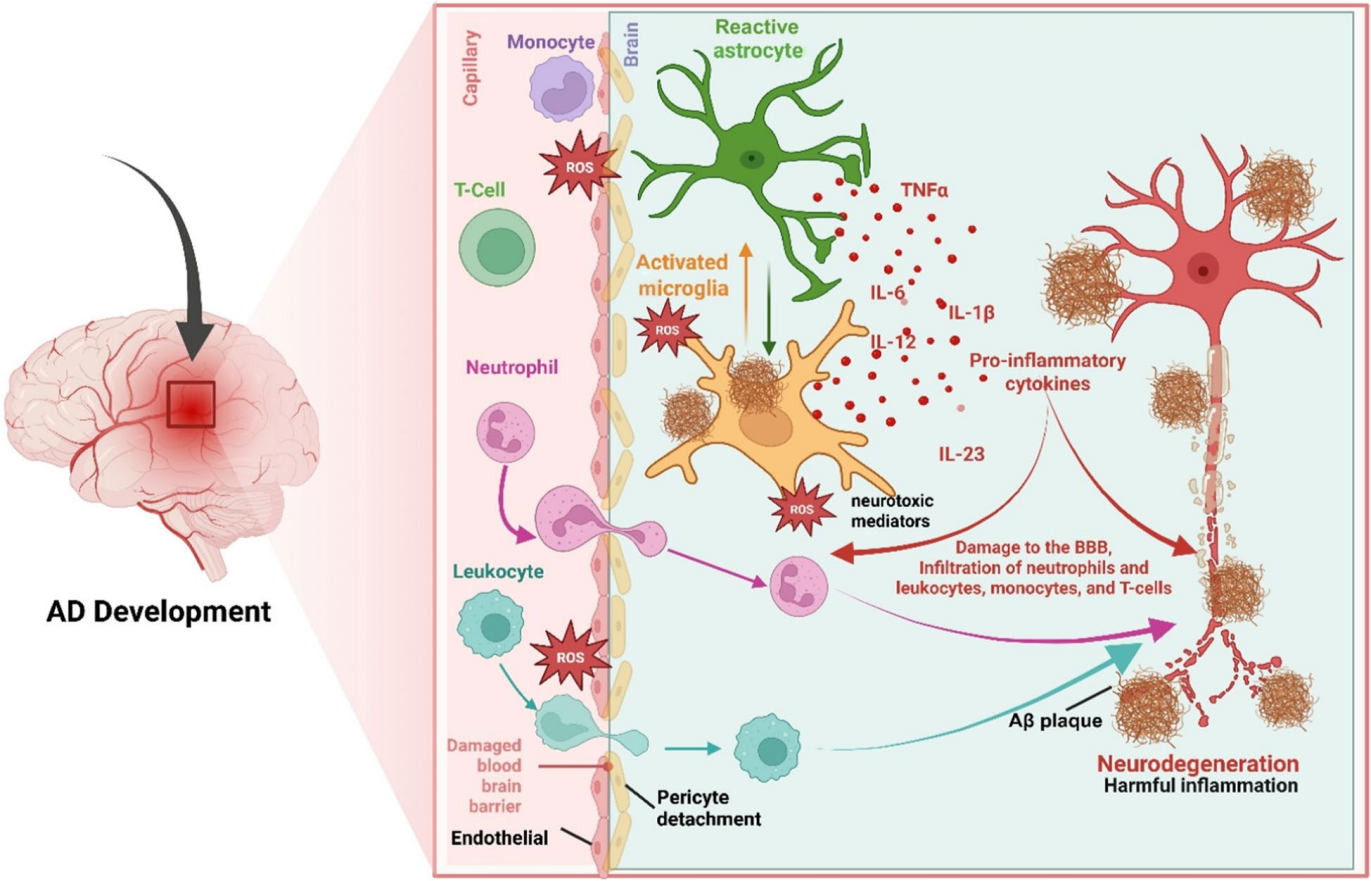
Neuroinflammatory response of glial cells to amyloid-β (Aβ) plaques in Alzheimer’s disease (AD). Activated microglia and astrocytes cluster around Aβ plaques, highlighting their dual roles in the disease process. Arrows extending from glial cells indicate the release of key pro-inflammatory cytokines (e.g., IL-1β, IL-6, TNF-α) and neurotoxic mediators such as reactive oxygen species (ROS). The schematic illustrates the progression of glial responses in AD, emphasizing the transition from a protective phase to a chronic, detrimental state. With sustained activation, glial cells begin to release pro-inflammatory cytokines and ROS, promoting chronic neuroinflammation, synaptic dysfunction, and neuronal damage. This transition contributes to the progressive neurodegeneration observed in AD

Astrocytes are equally important in the neuroinflammatory landscape of AD. Reactive astrocytes in AD worsen the inflammation and also lose their supportive homeostatic functions [23, 36, 37]. One recent work created a single-nucleus transcriptomic atlas of approximately 628,000 astrocytes from human brains across the spectrum of no pathology, early AD, and advanced AD [38]. The study identified several astrocyte subclusters with region-specific and stage-specific profiles. Strikingly, the proportion of homeostatic astrocytes versus reactive astrocytes varied by brain region (spatial heterogeneity) [38]. One subtype enriched with neurotrophic factors declines steadily as AD pathology worsens, suggesting a loss of supportive astrocytes. Another subtype increases in mid/late stages but then drops in end-stage AD, indicating an exhausted astrocyte response under chronic pathology [38]. A seminal study showed that activated microglia secrete factors (IL-1α, TNF-α, C1q) to induce A1 reactive astrocytes, a subtype that loses normal supportive functions and turns neurotoxic [39]. These astrocytes, especially the neurotoxic A1 subtype of astrocytes, secrete inflammatory mediators that promote neuronal injury [39]. Importantly, A1 astrocytes were found in postmortem brains of AD and other neurodegenerative diseases, indicating that this pathological astrocyte conversion occurs in human disease [39]. This finding highlights a detrimental crosstalk; microglia-driven inflammation can push astrocytes into a harmful A1 state, contributing to neurodegeneration. Expanding on this, the condition of chronic inflammatory stress can enhance Aβ amyloidogenesis. For instance, the microglia-derived cytokines IL-1α, TNF-α and C1q can induce astrocytes to overexpress β-secretase that in turn increases Aβ monomer peptide production [40]. This long-standing inflammation leads to a positive feedback: Aβ aggregates stimulate microglia which in turn discharge factors that are neurotoxic and promote the formation of more Aβ aggregates [16]. Over time, cumulative inflammatory damage has accelerated to a level that is debilitating and leads to progressive neurological deterioration (Fig. 2).

Neuroinflammation in AD patients is heterogeneous and stage-dependent [10, 41], which makes broad-spectrum immunosuppression a risky strategy. Transcriptomic analyses of human brains indicate that many immune-related changes occur early in the disease, even before noticeable clinical symptoms appear. For example, one work profiled approximately 80,000 single-nucleus transcriptomes from AD patient brains and found that each major cell type (i.e., neurons, microglia, astrocytes, etc.) contains a distinct disease-associated subpopulation when AD pathology is present [42]. Strikingly, the strongest disease-related gene expression changes are highly cell-type-specific and appear at early pathological stages, whereas genes upregulated in end-stage AD tend to be generic stress-response genes which are shared across cell types [42]. More specifically, microglia and astrocytes first activate defined inflammatory or phagocytic responses in the early stages of AD. Over time, as neurodegeneration becomes chronic, these responses shift into a generalized stress state that lacks specific functional programs. Moreover, microglia can also produce protective factors (e.g. TGF-β1) and support plaque clearance. Therefore, completely silencing immune cells early in disease could impair Aβ removal [43]. This aligns with longitudinal imaging studies. Biomarkers of neuroinflammation, such as cytokines or PET-detected microglial activation, can be detected years before cognitive decline, often paralleling amyloid deposition [44]. As the disease progresses, microglial activation becomes more pronounced and correlates with tau pathology and network dysfunction [33, 44]. This dual role, which has been referred here as a ‘double-edged sword’, implicates the importance of spatial targeting of neuroinflammation, i.e., inhibiting harmful inflammatory responses in the severely affected areas while maintaining beneficial immune functions in other regions. It is now becoming more obvious that any disease-modifying treatment for AD must incorporate management of neuroinflammation along with protein aggregation. Indeed, many AD drug candidates currently in clinical trials are aimed at inflammatory pathways [10], reflecting the recognition of neuroinflammation as a key therapeutic target. However, how to target neuroinflammation precisely and safely remains a key challenge. The temporal heterogeneity of neuroinflammation means that therapeutic timing is critical. More specifically, anti-inflammatory or immunomodulatory strategies might need to dial down harmful late inflammation without abolishing the early protective functions of microglia or astrocytes. To address the concern of silencing necessary immune responses, our proposed therapy aims to target specific inflammatory mediators that are known to be deleterious in AD such as IL-1β and TNF-α [45, 46], while preserving the beneficial functions of microglia and astrocytes. Conjugating anti-inflammatory payloads that selectively inhibit pro-inflammatory pathways, such as p38 mitogen-activated protein kinase (MAPK) inhibitors [47] and Toll-like receptor 4 (TLR4) antagonists [48], can help maintain the balance between protective and harmful immune responses. In addition, targeting microglial checkpoints (i.e., TREM2, APOE pathways) might boost their protective functions or prevent the switch to a toxic phenotype [31]. Likewise, inhibiting the signals that induce A1 astrocytes (such as IL-1α, TNF-α, C1q) could prevent supportive roles of astrocytes [39]. Future studies are needed to investigate the effects of this therapy on microglial phenotypes (e.g., DAMs) and astrocyte activation states to ensure that protective functions are not compromised.

Additionally, the heterogeneity and dynamism of AD pathology must be considered. Inflammatory signatures vary across patients and disease stages, necessitating a personalized approach. In early AD, characterized by mild cognitive impairment and low neuroinflammatory burden, therapies should aim to preserve protective immune functions, such as microglial plaque clearance and astrocyte neurotrophic support, potentially through enhancing anti-inflammatory cytokines like IL-10 or IL-4. In contrast, mid-to-late AD, marked by chronic pro-inflammatory states, requires targeted suppression of harmful mediators, such as IL-1β and TNF-α, in regions with dense Aβ plaques and activated microglia, as identified by translocator protein (TSPO)-PET imaging. Biomarker-guided strategies, including cerebrospinal fluid (CSF) cytokine profiles (e.g., elevated IL-1β/TNF-α indicating a need for suppression) and tau PET to correlate with pathology, can inform these decisions. In addition, patients harboring AD risk genes that affect microglial or astrocyte function may particularly benefit from targeted immunomodulation. Carriers of the *APOE* ε4 allele (who tend to exhibit greater plaque-related inflammation and BBB issues) and those with *TREM2* risk variants (who have impaired microglial activation and Aβ clearance) are good examples. Such genetic profiles can inform therapy choice. For instance, a TREM2-agonist antibody might be especially considered for a patient with a hypofunctional *TREM2* variant, aiming to boost microglial Aβ uptake. More broadly, genetic stratification aligns with precision-medicine approaches, suggesting that host factors (genotype, age, immune milieu) will determine the optimal type and the magnitude of immunomodulation [49]. Many risk gene effects appear stage-specific (e.g., inadequate early immune activation versus excessive late inflammation). Thus, understanding a patient’s genotype may guide whether to enhance or suppress immune responses at a given point [49].

Conversely, exclusion criteria should be defined to avoid treating patients unlikely to benefit or be prone to harm from immune-based therapies. A key exclusion consideration is the late stage of severe AD. Individuals in severe dementia stages (advanced tauopathy with extensive neuronal loss) are less suitable for immune-modulation. In these stages, the neuroinflammatory response may become less actionable. Microglia can enter a senescent or dystrophic state in end-stage pathology, and profound cortical tau burdens signal that damage is largely irreversible. In addition, immunosenescence in very elderly or advanced patients could blunt any therapeutic immune modulation. As such, most trials exclude patients beyond mild-to-moderate severity, recognizing that the window for modifying disease via inflammation is narrow.

While a detailed decision tree with specific clinical parameters is essential for clinical practice, it is beyond the scope of this conceptual review, which focuses on outlining general principles for immune modulation in AD.

Future research should also develop operational algorithms using longitudinal biomarker data to tailor therapies to individual patient profiles, ensuring precision in modulating neuroinflammation while maintaining beneficial immune functions. Biomarkers, such as CSF and cytokine profiles or PET imaging with TSPO ligands, can help stratify patients and monitor therapy response [50, 51].

## Limitations of current therapies and the case for targeted delivery

The reduction of amyloid plaque load by anti-Aβ mAbs has only a modest effect on cognitive decline [10], while these antibodies are associated with substantial inflammatory adverse events (Table 1). Significant number of patients receiving mAbs like aducanumab and lecanemab have ARIAs, which can be edema (ARIA-E) or microhemorrhages. Current evidence suggests that ARIA-E is an iatrogenic inflammatory response to these antibodies which bind to vascular Aβ, triggering the complement system. This process can lead to an inflammatory attack on the vessel wall and increased vascular permeability, resulting in fluid leakage into the brain parenchyma and edema [52, 53]. The results of leakage of vessels and microbleeds demonstrate that therapies intended to remove amyloid plaques can have adverse effect of increasing inflammation. Furthermore, the current mAbs bind to Fc receptors on microglia, which induce the phagocytosis of the plaques. Although this increase in immune activation is helpful for removing the plaque, it can also promote the production of proinflammatory cytokines in the microglial cells. In other words, conventional anti-amyloid immunotherapy with antibodies is a crude tool with respect to neuroinflammation: it targets one aspect of the pathology (amyloid deposition) at the cost of worsening the other (inflammation).

**Table 1:** Comparison of conventional vs. plaque-targeted anti-neuroinflammatory therapy in Alzheimer’s disease

Aspects	Conventional anti-inflammatory therapy	Plaque-targeted anti-inflammatory therapy
Target specificity	Non-specific; broadly targets general inflammatory pathways throughout the brain, such as cyclooxygenase (COX) enzymes implicated in widespread inflammation in Alzheimer’s disease (e.g., Ibuprofen) [58, 59].	Highly specific; selectively targets amyloid plaques, focusing directly on pathological aggregates of beta-amyloid (Aβ), reducing plaque-associated inflammation and neurotoxicity (e.g., antibody-based therapies such Aducanumab, Lecanemab) [60, 61].
Brain distribution	Widespread, non-targeted distribution; limited penetration due to blood–brain barrier (BBB), often insufficient to reach deeper brain regions effectively impacted by Alzheimer’s disease pathology [62, 63].	Precisely localized distribution; effectively crosses the BBB through advanced delivery methods like antibody drug conjugation, resulting in high drug concentrations specifically at amyloid plaque locations deep within brain tissue [64, 65].
Local drug concentration	Typically, low concentration within the brain due to inadequate BBB penetration, rapid systemic clearance, and non-targeted distribution, compromising effectiveness in alleviating localized neuroinflammation and neuronal damage [62, 63, 66].	High, sustained concentration precisely at amyloid plaques, significantly enhancing localized bioavailability, allowing direct modulation of plaque-associated neuroinflammation and neuronal protection [60, 61, 64, 65].
Systemic exposure & side effects	Significant systemic exposure, with increased risk of widespread adverse effects, including gastrointestinal issues (e.g., bleeding, ulcers), renal impairment, cardiovascular risks, and generalized immunosuppression due to broad anti-inflammatory activity (e.g., NSAID-induced complications) [58, 67, 68]	Minimized systemic exposure due to targeted localization, significantly lowering the risk of systemic side effects; primarily mild infusion-related reactions, significantly reducing the risks of gastrointestinal, renal, or cardiovascular side effects common with non-specific therapies [60, 61, 64, 65].
Efficacy in AD	Limited efficacy due to non-specific action and insufficient drug concentration at pathological sites; resulting in marginal or no significant improvement in cognitive function or slowing of disease progression in clinical studies [66, 69, 70].	Higher efficacy due to specific targeting of amyloid plaques, leading to direct reduction in plaque load, alleviation of local inflammation, preservation of neuronal integrity, and clinically meaningful improvements in cognitive function and reduction of disease progression [60, 61, 71–73].
Dosing requirements	Frequent, high-dose regimens required due to short half-life, poor brain penetration, rapid systemic metabolism, and non-specific action (e.g., multiple daily doses needed to maintain therapeutic effects) [62, 63, 66].	Lower and less frequent dosing required; targeted therapies (e.g., monoclonal antibodies) allow prolonged retention at amyloid sites, typically administered as monthly intravenous infusions or injections, substantially improving patient compliance [60, 61, 64, 65].

However, efforts of broad-spectrum anti-inflammatory treatment in AD have been largely unsuccessful. Many clinical trials with NSAIDs or other anti-inflammatory drugs in patients with established AD or dementia have not led to any therapeutic benefit [54–56]. There are several possible explanations for these failures: the side-effects are typical for long-term use of systemic NSAIDs, and the agents are often poorly effective at the maximum tolerable BBB penetration; non-specific immunosuppression may hinder beneficial immune functions such as microglial amyloid clearance. For example, a small AD trial with anti-cytokine biologics like anti-TNFα antibodies was only partly effective, likely because these agents are unable to target specifically affected brain regions or reach sufficient CNS concentrations [57].

Collectively, these findings suggest that appropriate administration of anti-neuroinflammatory treatment in AD is a function of spatial directionality and temporal proximity to the affected region, and that simply flooding the brain or body with anti-inflammatory agent is neither safe nor effective. Conventional antibody-based anti-amyloid therapies effectively target amyloid deposits but may inadvertently exacerbate neuroinflammation, representing a significant limitation. A targeted approach, linking immunomodulatory therapies directly to disease hallmarks such as Aβ plaques, could precisely disrupt neuroinflammatory processes mediated by activated microglia and astrocytes, minimizing off-target effects and preserving the overall brain immune function.

## A next-generation targeted therapeutic strategy: development of a plaque-specific sdAb

Central to this strategy is an sdAb engineered to bind with high specificity to aggregated Aβ, particularly the fibrillar forms found in plaques. Those antibodies are the smallest functional antibody fragments (10–15 kDa), consisting of a single monomeric variable heavy chain domain. Despite their compact size, sdAbs exhibit remarkable affinity and specificity for their targets [74]. Derived from heavy-chain-only antibodies naturally occurring in camelids, these molecules can be humanized for therapeutic applications. The sdAbs selected for AD therapy should be able to recognize a conformational epitope unique to Aβ fibrils or oligomers, enabling precise targeting of amyloid plaques with minimal off-target interactions. A key feature of sdAbs is their lack of an Fc region, unlike conventional antibodies, which prevents Fc-mediated immune responses such as complement activation.

In addition to their specificity, sdAbs are particularly suited for CNS drug delivery due to their small size and single-domain structure, enhancing tissue penetration and potentially improving BBB permeability compared to conventional antibodies [75, 76]. Additionally, their small size is suitable for accessing the sterically constrained epitopes which are usually located in close proximity to the dense structure of amyloid plaques. SdAbs display high stability and can be easily modified or conjugated with other molecules and are currently used as therapeutic and imaging agents in neurological disorders [77, 78]. Therefore, a high-affinity anti-Aβ amyloid sdAb is a suitable carrier to transport the therapeutic agent to the affected brain.

## Anti-inflammatory payload

sdAbs can serve as a carrier for diverse anti-inflammatory payloads, significantly enhancing their therapeutic impact against neuroinflammation in AD. Key payload classes include small-molecule agents, biologics, RNA-based therapeutics, peptide and proteins, and nanoparticle (NP) systems, each offering unique mechanisms and therapeutic advantages (Table 2).

**Table 2:** Types of anti-inflammatory payloads

Payload type	Examples	Mechanism of action	Advantages	Challenges
Small molecules	MCC950 (NLRP3 inhibitor), NSAIDs (e.g., Ibuprofen), Steroids (e.g., Dexamethasone) [79].	Inhibit inflammatory pathways (e.g., NLRP3 inflammasome activation, COX inhibition, glucocorticoid receptor-mediated inflammation reduction) [79, 80].	High potency and stability; ease of synthesis and modification; broad availability and affordability [62, 79].	Limited BBB penetration, potential off-target effects, dosing control issues, systemic side effects (e.g., gastrointestinal, cardiovascular risks for NSAIDs; metabolic and immunosuppressive effects for steroids) [62, 79].
Biologics: Cytokines	IL-10 (anti-inflammatory cytokine), antibodies (e.g., Aducanumab) [60, 81, 82].	Promote anti-inflammatory signaling; regulate immune response in brain; specifically target pathological proteins [60, 81, 82].	Sustained receptor engagement, specific targeting, high therapeutic efficacy [60, 81, 82].	Stability of linker and biologics, immunogenicity, challenging BBB penetration, cost and complexity of manufacturing [60, 81, 82].
Biologics: RNA-based payloads	siRNA, miRNA mimics or inhibitors [83]	Silence pro-inflammatory genes or modulate gene expression through RNA interference or regulation [83].	High specificity and potency, capability to target multiple disease pathways simultaneously [83].	Stability and degradation, efficient delivery and targeting challenges, off-target effects [83].
Biologics: Neurotrophic factors	Anti-inflammatory peptides (e.g., Humanin), neurotrophic factors (e.g., BDNF) [84–86].	Modulate signaling pathways, reduce inflammation, support neuronal survival and function [84–86].	High specificity, efficacy in modulating complex pathways [84–86].	BBB penetration challenges, rapid degradation, immunogenicity, delivery difficulties [84–86].
Nanomedicine-based payloads	Nanoparticle carriers (liposomes, polymeric nanoparticles) [65, 87].	Enhance targeted drug delivery, payload protection, controlled release [65, 87].	High payload capacity, targeted delivery, controlled release, improved bioavailability [65, 87].	Size-related issues for BBB penetration, stability in physiological conditions, control of dosing and release kinetics, complex synthesis processes [65, 87].

*BDNF* brain-derived neurotrophic factor

## Small-molecule payloads

Small molecules provide potent, specific inhibition of inflammatory pathways and exhibit favorable chemical stability. Examples include inhibitors targeting microglial signaling or cytokine production: NLRP3 inflammasome inhibitors like MCC950 suppressing IL-1β release from microglia; p38 MAPK inhibitors, such as MW150, reducing pro-inflammatory cytokines (e.g., IL-1β, TNF-α) and improving memory deficits in AD models [88]; and TLR4 antagonists, such as TAK-242 or IAXO-101, shifting microglia toward an anti-inflammatory phenotype while enhancing cognition in preclinical studies [89, 90]. Small molecules are typically lipophilic and membrane-permeable, potentially enabling diffusion into glial cells upon release. Their non-protein nature also confers low immunogenicity [91, 92]. However, conjugation requires a stable yet cleavable linker (discussed below) to prevent premature release. By delivering these agents focally to plaques, we aim to enhance their therapeutic index. Critical considerations include ensuring conjugate stability in circulation and confirming that the drug retains bioactivity after release at the target site.

## Biologic payloads

Biologic payloads can directly engage cellular pathways to resolve inflammation. Within biologics, diverse therapeutic modalities have emerged, expanding the potential of nanobody-mediated approaches against AD. RNA-based payloads, including small interfering RNA (siRNAs) and microRNAs, offer precise gene-silencing capabilities targeting neuroinflammatory pathways [83]. Peptides and proteins provide versatile means to modulate disease processes directly, such as reducing amyloid pathology or inflammatory signaling [84–86].

Anti-inflammatory cytokines are particularly promising. IL-10, a potent immunoregulatory cytokine, inhibits pro-inflammatory microglial outputs and protects astrocytes from excessive activation [81]. Applying local delivery of IL-10 to plaque sites could suppress cytokine production and promote homeostasis. However, prolonged IL-10 exposure might impair Aβ plaque clearance [93], necessitating careful control of dosing and timing. IL-4 presents another candidate, promoting an M2 (protective) microglial phenotype, enhancing Aβ fibril phagocytosis, and stimulating neurotrophic factor release [94]. A single intracranial injection of IL-4 in an AD mouse model improved memory performance, underscoring its therapeutic potential [95]. These cytokines can be attached to the nanobody via genetic fusion or chemical linkage, with flexible peptide linkers ensuring preserved bioactivity (e.g., enabling receptor binding on glial cells). Unlike small molecules, biologic payloads may provide sustained action. For example, the antibody-IL-10 fusion could continuously modulate nearby glia while bound to a plaque. In summary, biologics can be used to modulate glial responses with better control than using a molecule to inhibit an enzyme.

## NP systems

In the NP system, the anti-Aβ fibril sdAb serves as a targeting ligand on the surface of an NP, such as a liposome, polymeric particle, or micelle, which encapsulates the therapeutic payload. NPs offer distinct advantages: they can carry larger or multiple payloads, protect sensitive biologics from degradation, and enable delivery of nucleic acids. For instance, lipid nanoparticles (LNPs) formulated to deliver siRNA specific to the microglial transcription factor PU.1 (*SPI1*) or NLRP3 have been reported to alleviate neuroinflammation and pathology in AD models [96]. When administered into the CSF, such LNPs preferentially enter activated microglia, silencing pathogenic genes. Similarly, polymeric NPs loaded with natural anti-inflammatory compounds (e.g., curcumin) have shown promise in preclinical AD studies [97]. By coating NPs with the anti-Aβ sdAb, they can accumulate at plaques and release their cargoes within the inflammatory microenvironment. NPs also support modulators of solubility or combination therapy (for example, a steroid together with a cytokine). However, there are some issues: to reach the brain, the size of NP must be optimized (large particles may not be able to diffuse freely); the material should be biodegradable to avoid accumulation effects. These risks can be reduced by using stealth coatings (e.g., polyethylene glycol) and biocompatible materials. Also, the sdAb-NP association should be stable while permitting accurate plaque recognition during targeting. When it comes to efficacy, NPs may give higher therapeutic effect compared to single sdAb-drug conjugate due to the ability to saturate the inflamed regions. However, the sdAb-NP may have increased complexity in the manufacturing and the characterization of the product.

## Comparison of the payload modalities for targeting neuroinflammation

It is imperative to conduct a comparison of small molecules, cytokines, and nanoparticles for payload to treat neuroinflammation in AD. These modalities are evaluated based on key criteria: efficacy in modulating neuroinflammation, stability and half-life, target engagement, and diffusion and tissue penetration.

Efficacy in mitigating neuroinflammation is a primary consideration, typically assessed by the reduction of microglial inflammatory markers such as IL-1β, TNF-α, and iNOS (inducible nitric oxide synthase) and improvements in neuronal health in AD models. Small molecules, particularly kinase inhibitors, have shown robust preclinical efficacy, significantly lowering cytokine levels and amyloid plaque burden. In contrast, cytokines like IL-4 and IL-10 promote glial repolarization toward a neuroprotective phenotype and thereby attenuate inflammatory responses [98].

The payload’s stability and half-life are important for optimal therapeutic effects. Chemical stability and extended half-life are generally high for small molecules when they are conjugated to an sdAb, which is therefore suitable for targeted delivery [99, 100]. Many biologics, such as cytokines, require optimization of the structure, such as fusion to an Fc domain to enhance stability and increase the time they remain active in the body [100]. By encapsulating payloads in nanoparticles, the half-life of the drugs is increased, and they are protected against degradation; but particle pinocytosis may be unfavorable, resulting in a reduced particle residence time in the target tissues [100, 101]. These differences stress the necessity of tailoring stability for the desired duration of action.

Target engagement distinguishes the modalities in their interaction with biological targets. Biologics, such as cytokines, can actively bind cellular receptors even when tethered to the sdAb, enabling sustained signaling [102]. For instance, an sdAb–IL-10 conjugate may engage glial receptors to trigger anti-inflammatory pathways without requiring dissociation [81, 103]. Small molecules, however, typically must dissociate from the conjugate to penetrate cells and inhibit intracellular targets, such as kinases [104]. This difference suggests that biologics may be useful when long-lasting receptor-mediated effects are desired, while small molecules may be more appropriate for regulating intracellular events.

It is also essential that the payload can be effectively delivered to the target site through optimal diffusion and penetration of the tissues. The small molecules that are released from the sdAb conjugate exploit their small size and lipophilicity to cross the cell membranes easily and penetrate through tissues, thus ensuring a high distribution. The diffusion range of larger biologics, cytokines, is more restricted; however, when they are released extracellularly, they can affect numerous adjacent cells and thereby increase their regional impact. The size and design of nanoparticles determine how long they remain at the binding site before degradation. This increases their local effect but may also limit their penetration into deeper tissues [105, 106].

## Payload release mechanisms

A critical consideration of this therapeutic design is the mechanism by which the payload detaches from the sdAb carrier after localizing it to an amyloid plaque. Two primary strategies are considered: triggered (active) release and sustained (conjugated) action.

## Triggered release

**Fig. 3:**
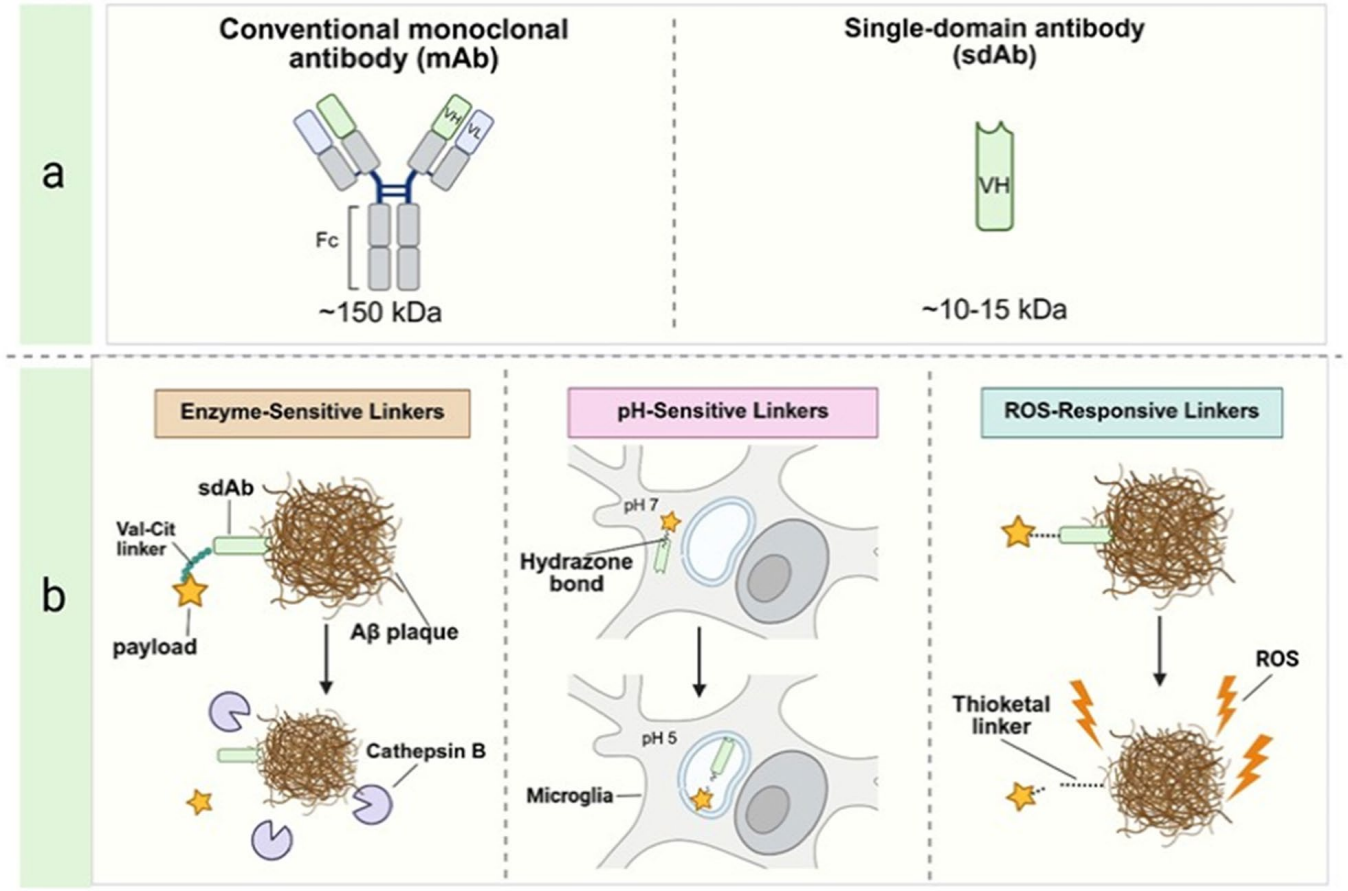
Structural comparison of single-domain antibodies (sdAbs) and monoclonal antibodies (mAbs), and targeted payload release mechanisms using stimuli-responsive linkers. **a** A side-by-side schematic compares the structural and functional characteristics of a conventional mAb (~ 150 kDa) and an sdAb (~ 10–15 kDa). The mAb is shown with two heavy and two light chains forming a Y-shaped structure with an Fc region, while the sdAb is depicted as a compact, single variable heavy chain domain lacking an Fc region. Labels highlight the sdAb’s reduced molecular weight and enhanced ability to access sterically restricted epitopes on amyloid-β (Aβ) plaques. **b** Left, enzyme-sensitive linkers. An sdAb-payload conjugate is shown bound to an Aβ plaque. Nearby, plaque-associated enzymes such as cathepsin B are illustrated as small Pac-Man-like figures. The linker connecting the payload to the sdAb is depicted as a short chain, labeled with a "cleavage site". Upon enzymatic recognition, the linker is cleaved, releasing the payload into the local environment. Middle, pH-sensitive linkers. Inside a microglial cell, an sdAb conjugate is shown enclosed within an endosome, labeled "pH 5.0". The linker between sdAb and payload (illustrated as a zigzag line) is cleaved under acidic conditions, releasing the payload into the intracellular space. Labels identify the "endosome" and "pH-sensitive linker". Right panel, reactive oxygen species (ROS)-responsive linkers. Near the Aβ plaque, an sdAb conjugate is exposed to a ROS environment, indicated by lightning bolt or star symbols. A dashed line represents the ROS-sensitive linker, which degrades upon oxidative exposure, triggering payload release. Labels indicate "ROS" and "ROS-responsive linker"

Triggered release (discussed below) utilizes cleavable linkers to offload the payload from the sdAb exclusively within the inflammatory microenvironment, enhancing specificity, minimizing off-target effects, and optimizing delivery to pathological regions (Fig. 3) [107].

Enzyme-sensitive linkers. These linkers take advantage of the increased protease activity in the AD-affected brain regions, which have higher levels of enzymes including matrix metalloproteinases that are released by reactive astrocytes or cathepsins that are secreted by activated microglia. Peptide-based linkers that are designed as substrates for these proteases allow for controlled and site-specific payload release. A canonical example is the Valine-Citrulline dipeptide linker, which is used extensively in antibody–drug conjugates (ADCs) owing to its stability in circulation and sensitivity to cleavage by lysosomal cathepsin B [108]. In AD therapy, when microglia phagocytose sdAb-drug conjugates that are attached to opsonized Aβ aggregates, cathepsin-mediated cleavage of the linker within the lysosomes delivers the payload intracellularly. Furthermore, linkers that respond to extracellular proteases, including ADAM17 (A disintegrin and metalloproteinase domain-containing protein 17), can deliver the payload near the plaques if the enzymes are secreted or are highly expressed by glial cells during inflammation. Thus, leveraging the knowledge of the enzymatic gradient in the AD brain, the design of this approach is optimized to establish a sophisticated in situ delivery system.

pH-sensitive linkers. These linkers are based on the acidic pH of endosomal compartments of the target cells. Steroidal conjugates bearing acid labile bonds like hydrazones or Schiff bases are chemically stable at neutral pH but break down at acidic pH. For example, a steroid conjugate linked by a hydrazone moiety could deliver its payload to microglial endosomes after the pH drop, causing the release of an anti-inflammatory agent inside the cell. This mechanism can be validated in vitro by conjugate testing against a pH gradient and evaluating the payload release, effective in the acidic environment of inflamed tissues [106, 109].

ROS-responsive linkers. Chronic neuroinflammation in AD generates elevated ROS, which ROS-responsive linkers exploit for targeted payload release. Links that contain thioketal or selenium bonds break down in the presence of oxidative stress and thus can deliver the payload to regions of high ROS, including degenerating plaque regions. This increases the specificity of the delivery to the oxidative damage that is characteristic of the AD pathology. The effectiveness of ROS-responsive conjugates in inflammation-targeted delivery systems points to their potential for use in AD therapeutics [110].

## Sustained action

Sustained action (discussed below), on the other hand, uses non-cleavable linkers or fusion proteins to anchor the payload to the sdAb at the inflammation site, sustaining the therapeutic effect and targeting the delivery to the area. This approach is ideal for biologic payloads that are able to work when conjugated [111].

Sustained conjugation creates a localized therapeutic depot at the plaque site which increases the duration and specificity of action. An example is the anti-fibronectin Ab fused to IL-10, which accumulates at amyloid plaques and forms a depot of IL-10 that can signal to microglia and astrocytes [111, 112]. Since the sdAb remains bound to fibrillar Aβ, the accompanying IL-10 can interact with its receptors on glial cells in the plaque area and sustain the anti-inflammatory microenvironment. As sdAbs have half-lives of days to a week in the brain, this approach maintains the duration of therapeutic activity. Restriction of payload diffusion is achieved through sustained conjugation and thus limits the effects to the plaque site and decreases systemic exposure and the associated risks [111].

Pharmacokinetic analysis becomes simpler with sustained conjugation as the sdAb-payload complex acts as a single unit in tracking and distribution studies. For payloads that need cytosolic access, endosomal escape mechanisms may be employed to allow the release of the payload from the endosome. Therefore, even without the linker cleavage, the sustained conjugation leads to the targeted delivery of the entire complex through the cellular uptake, which may increase the efficacy of plaque clearance.

## Strategies for therapeutic delivery

**Fig. 4:**
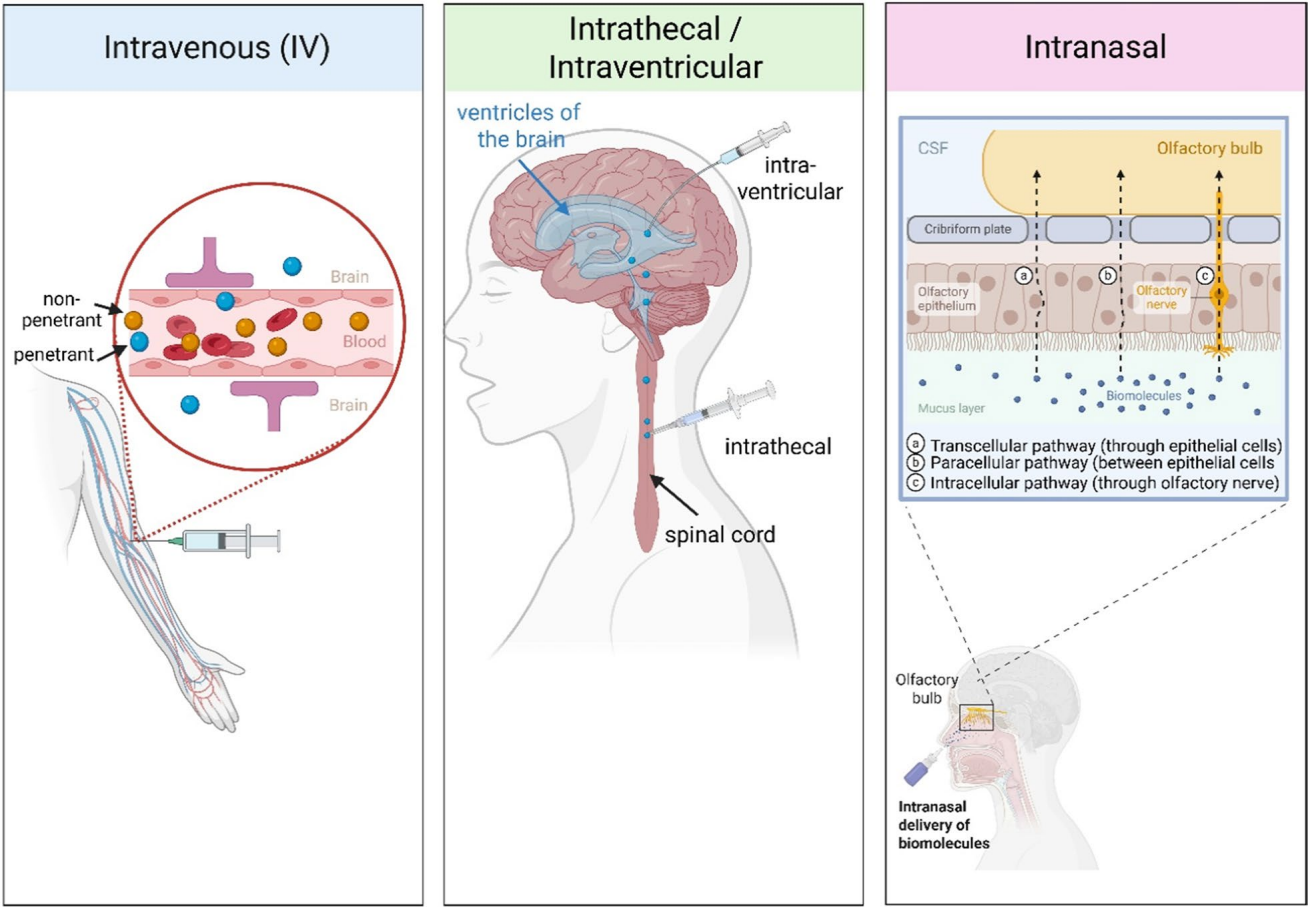
Routes of administration for delivering single-domain antibody (sdAb)-based therapeutics to the central nervous system in Alzheimer’s disease. Left, intravenous (IV) administration delivers the therapeutic agent systemically through the bloodstream. Limited permeability across the blood–brain barrier (BBB) is highlighted, emphasizing the challenge of achieving sufficient brain exposure via this route. Middle, intrathecal or intraventricular injection is shown, delivering the agent directly into the cerebrospinal fluid (CSF), allowing for diffusion into the brain parenchyma and enhanced CNS bioavailability, bypassing the BBB. Right, intranasal administration is illustrated via delivery through the nasal cavity, with arrows indicating nose-to-brain transport pathways along the olfactory and trigeminal nerves, providing a non-invasive route to target brain regions

Delivering therapeutic antibodies to the brain is not a straightforward process due to the existence of the BBB, which hinders large molecules such as antibodies from entering the brain. Accurate delivery of anti-Aβ fibril sdAb-payload conjugates to the sites of neuroinflammation is crucial for the efficacy in AD. This section discusses different administration routes, including systemic (intravenous [IV]), intrathecal or intraventricular (into the CSF), and intranasal administration of these conjugates along with the available means of increasing their penetration into the brain (Fig. 4). Each approach has its advantages and disadvantages, and all need to be considered in light of their use in AD therapy.

The conventional route of administration of anti-Aβ antibodies (aducanumab, lecanemab) in clinical settings is IV administration, which allows repeated dosing and is well established in clinical practice. However, the BBB is a significant impediment to the brain uptake and only a small fraction reaches the CNS [113]. To address this limitation, high doses are frequently used in clinical trials, though this approach increases the risk of systemic side effects.

Advanced strategies, such as the "Trojan horse" approach, which uses bispecific antibodies that target both Aβ and BBB receptors, are under investigation to improve brain delivery. Moreover, evidence suggests that AD pathology may modulate the BBB and that breakdown of the barrier and impaired perivascular clearance in affected regions may facilitate antibody uptake [114, 115]. However, active transport mechanisms are desired to achieve greater therapeutic concentrations in the brain.

Direct administration of the sdAb-payload conjugate into the CSF via intrathecal or intraventricular injection avoids the BBB, placing the therapeutic agent directly in the vicinity of the brain’s extracellular space. As a more invasive method, this route can lead to higher central drug concentrations while keeping systemic exposure low and, therefore, may have a lower incidence of peripheral side effects. Intrathecal infusion is currently used in the treatment of neurodegenerative diseases, including antisense oligonucleotides for spinal muscular atrophy and AD tau knockdown [116]. In AD models, the distribution and extent of conjugate accumulation in cortical and hippocampal plaques are key factors for efficacy. Limited penetration into deeper brain structures and potential adverse effects, such as meningeal inflammation after repeated CSF injections, require careful monitoring.

Intranasal administration provides a non-invasive alternative for CNS delivery by leveraging neural pathways, including the olfactory and trigeminal routes, which connect the nasal mucosa to the brain [117]. Studies have demonstrated that large molecules, such as antibodies, can reach the rodent brain through this pathway, likely via paracellular transport along olfactory nerve fibers [118]. In AD models, intranasal delivery of therapeutic agents, including nanoparticles, has shown promise in reducing Aβ burden [119]. If the sdAb-payload conjugate is formulated as a mucoadhesive spray, the patient can self-administer it, ensuring more stable brain levels, which is important for chronic treatment. Despite its advantages, this approach faces challenges, including stability in the nasal environment and achieving an effective dosage to modulate neuroinflammation. Absorption enhancers, permeation peptides, or linkage to small nanoparticles may improve nose-to-brain delivery and enhance therapeutic effects.

## Strategies for enhancing BBB penetration of therapeutic antibodies

Effective delivery of therapeutic agents to the brain remains a critical challenge in treating neurological disorders due to the restrictive nature of the BBB. To address this challenge, advanced strategies are being developed. Among these, receptor-mediated transcytosis (RMT) stands out as a state-of-the-art approach, leveraging endogenous transport mechanisms to facilitate brain entry. This section explores RMT as a strategy for BBB penetration, detailing its mechanisms, design considerations, and potential applications in preclinical evaluation [120, 121].

**Fig. 5:**
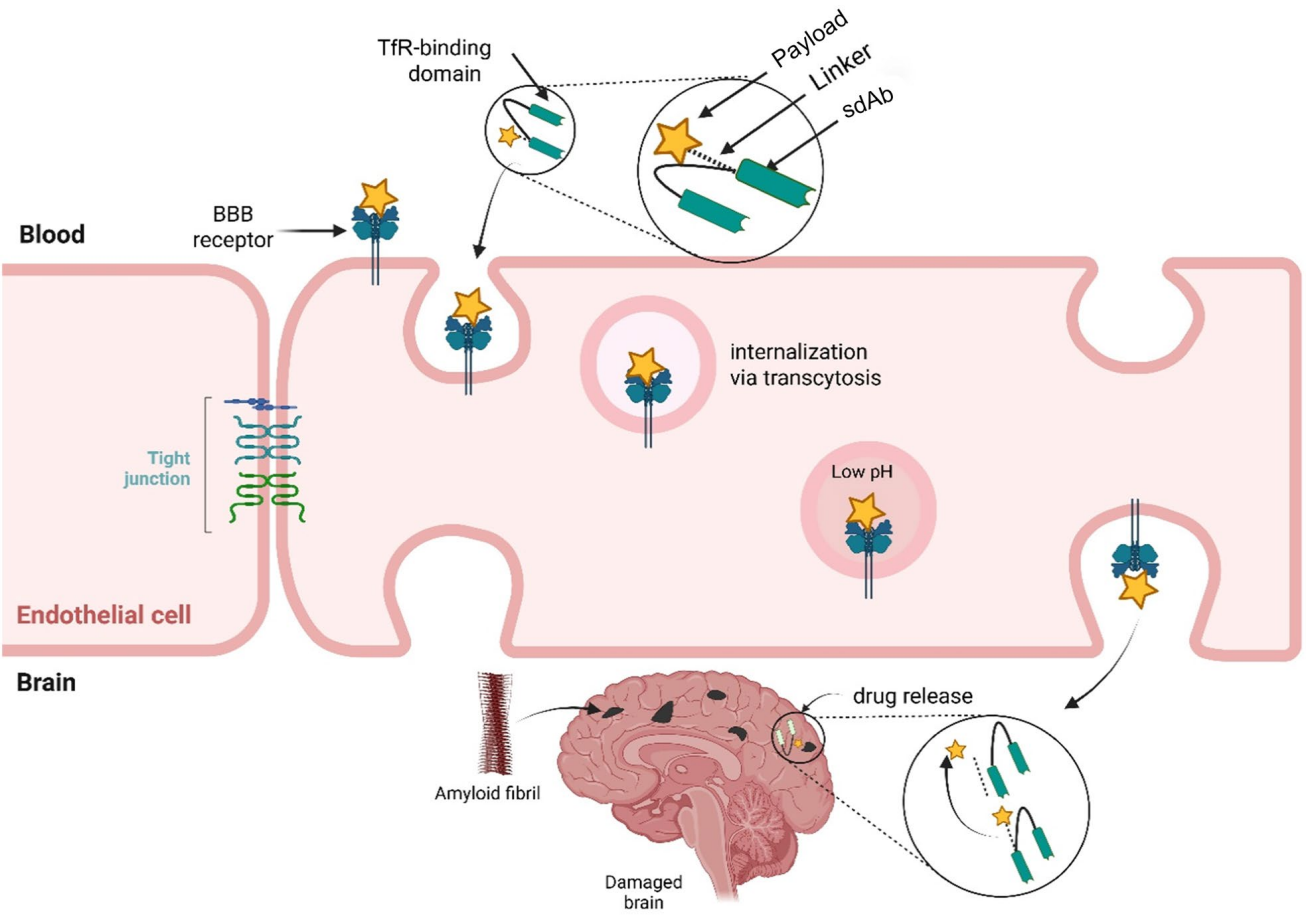
Receptor-mediated transcytosis of single-domain antibody (sdAb)-payload conjugates across the blood–brain barrier (BBB). This schematic illustrates a strategy for delivering sdAb-based therapeutics to the brain via receptor-mediated transcytosis. The sdAb-payload conjugate is shown binding to a receptor on the luminal surface of a brain endothelial cell, such as the transferrin receptor (TfR), which facilitates internalization through endocytosis. The complex is transported across the endothelial cell and released on the abluminal side into the brain parenchyma. Once within the CNS, the sdAb-payload conjugate diffuses to target amyloid-β (Aβ) plaques. This mechanism enables non-invasive delivery of therapeutic agents across the BBB, leveraging physiological transport pathways to enhance CNS exposure

RMT strategies focus on receptors expressed on the luminal surface of brain endothelial cells, including the transferrin receptor (TfR), insulin receptor, and IGF-1 (insulin-like growth factor-1) receptor. These receptors facilitate the transport of their respective ligands across the BBB. To harness this mechanism, the anti-Aβ sdAb can be re-engineered as a bispecific antibody, with one binding site targeting a transport receptor and the other directed against Aβ plaques. Alternatively, the sdAb can be conjugated to a ligand that binds these receptors (Fig. 5). Upon receptor engagement, the antibody-receptor complex is internalized and transported across the endothelial cell layer, releasing the therapeutic payload into the brain parenchyma, where the anti-Aβ component can bind to fibrillar Aβ plaques [120].

A key design parameter in RMT is the affinity of the antibody or ligand to the transport receptor. It has been found that ultra-high affinity to receptors like TfR can lead to the antibody being trapped within the endothelial cells and unable to leave for the brain [122].​ In order to tackle this problem, moderate affinity variants are used, which underscore a balance between efficiency of transcytosis and ease of release into the brain parenchyma. Furthermore, the design should avoid interference of the therapeutic payload with the transcytosis process, so that the transport and therapeutic components remain functional.

In addition to antibodies, RMT can improve the delivery of NP-based therapeutics. NPs can be coated with ligands targeting TfR or apolipoprotein E mimetics to facilitate their transcytosis across the BBB. This enables the delivery of a variety of payloads from small molecules to nucleic acids or other biologics encapsulated within the NPs, thus extending the therapeutic scope of RMT [120, 121].

## Additional considerations for developing a targeted immunotherapy for AD

The design of a targeted immunotherapy for AD using an sdAb-payload conjugate comes with many important considerations to ensure that the therapy is not only effective but also safe for the patient. This section (see below) discusses other important factors that include safety and immunogenicity, the preclinical and clinical translation pathway of the candidate immunotherapeutics, and the ethical and regulatory considerations (Table 3). All these elements are collectively used to help design and evaluate this novel therapeutic approach that regulates the neuroinflammatory response in AD with minimal risk.

**Table 3:** Additional considerations for development

Consideration	Key points	Mitigation strategies
Safety	-Microglial suppression risk: Microglia play a central role in AD pathology, and excessive suppression may exacerbate neurodegeneration [123] -Off-target effects: Unintended impact on other immune cells in the brain	-Dose optimization: Gradual dose escalation to find therapeutic window -Targeted delivery: Strategies (e.g., BBB-shuttles) that confine drug action within diseased regions
Immunogenicity	ADA formation: Therapeutic proteins (e.g., single-domain antibodies) can trigger anti-drug antibodies, impacting efficacy and safety -Prolonged use concerns: Chronic administration in AD patients can amplify immunogenic risk	-Humanized sdAb: Reduction of exogenous epitopes to limit immunogenicity -Regular immunomonitoring: Assess potential ADA development during long-term treatment
Translation pathway	-Robust preclinical models: Multiple transgenic AD mouse models needed to capture heterogeneity (amyloid, tau, inflammation) -Validation of mechanistic endpoints: Microglial and neuronal markers, cognitive outcomes	-GLP toxicology: Regulatory-compliant toxicology studies prior to IND -Multimodal biomarkers: Use imaging (PET/MRI) and fluid biomarkers (CSF, blood) to confirm on-target effects
Ethics/regulation	-Specificity validation: Particularly crucial for therapies altering immune responses in the CNS -Patient selection: AD is heterogeneous; advanced disease may differ significantly from early AD	-Regulatory engagement: Early communication with agencies (FDA, EMA) for clarity on data requirements -Stringent inclusion criteria: Clear patient stratification in clinical trials

*ADA* anti-drug antibodies, *GLP* good laboratory practice, *IND* investigational new drug

## Safety considerations

A central safety concern in this therapeutic strategy is the potential for excessive suppression of microglial function. Microglia are essential for brain homeostasis, performing roles such as clearing cellular debris and defending against pathogens. Overly aggressive inhibition could impair these functions, potentially exacerbating neurodegeneration or increasing infection risk. Thus, the therapy is designed to achieve immune modulation rather than complete microglial suppression, targeting pathological inflammation while preserving baseline microglial activity [24, 124].

Another critical safety aspect is the potential neurotoxicity of the payload. If the small molecule payloads are released inappropriately, they may affect other brain cells. In the case of nucleic acid-based payloads such as siRNA or gene therapy vectors, off-target gene silencing is another potential risk. Pharmacology studies need to be conducted in at least two animal species, including mouse and disease-relevant non-human primate models. Neurobehavioral and histopathological endpoints will be utilized to determine the therapeutic window and potential adverse effects [24, 124].

In addition to these concerns, the immunogenicity of the nanobody conjugate must be carefully evaluated, as repeated administration could lead to the development of anti-nanobody antibodies, potentially neutralizing the therapy or causing allergic reactions. To mitigate this concern, humanized nanobodies or those derived from human sequences can be used to reduce immunogenicity [76, 125, 126]. Furthermore, the choice of linker between nanobody and the payload is crucial, as it must be stable in circulation but cleavable at the target site to release the payload effectively [125]. Stability studies (formulation studies) and in vitro release assays should be conducted to optimize the linker design [125–127]. Moreover, the potential for off-target effects of the payload must be assessed, especially if the payload has systemic effects [126]. For instance, if a cytokine is used as a payload, its pleiotropic effects must be considered, and the dose must be carefully titrated to achieve local concentrations that are therapeutic without causing systemic toxicity [125, 126].

A comprehensive review article highlights key safety-related properties of nanobodies for brain disorders [76]. The authors stated that even though single-domain nanobodies are extremely small, they maintain high antigen affinity and specificity, which helps minimize off-target binding [76]. Because of the lack of Fc region, nanobodies do not trigger Fc-mediated immune activation in the brain, potentially avoiding inflammatory side effects that full immunoglobulin G (IgG) antibodies can cause [76, 125]. They concluded that the small size and high specificity of these nanobodies confer improved tissue or BBB penetration and reduced off-target toxicity compared to conventional antibodies [76]. One more work, on the other hand, emphasized the rapid systemic clearance of nanobodies, which can reduce prolonged exposure to peripheral organs and their high target selectivity, which lowers the risk of off-target effects in the body [126]. Further preclinical and clinical evaluations are required to ensure safety in long-term administration, specifically for any unintended interactions within CNS tissue.

## Immunogenicity risks

Immunogenicity is a major issue with biologic therapies, particularly those involving novel antibody conjugates or fusion proteins, as repeated dosing may induce immune responses [128]. The induction of anti-drug antibodies (ADAs) could diminish the efficacy of the therapy or provoke adverse reactions, necessitating strategies to minimize immunogenicity. To address this, sdAbs can be constructed with a fully human or humanized IgG backbone to reduce its recognition as foreign by the immune system. However, incorporating an Fc region—such as in a bispecific antibody—requires careful consideration, as its immune-activating properties may heighten the risk of ARIA in AD. To mitigate this, the Fc region can be modified to silence effector functions, thereby limiting its potential to trigger ARIA while preserving the structural benefits of the construct. For small-molecule payloads, immunogenicity is typically low; however, the linker component could generate novel epitopes if it interacts with serum proteins to form haptens. ADCs may exhibit higher immunogenicity than unconjugated antibodies due to their non-natural components (linkers or synthetic peptides) [91]. Preclinical studies need to include serial serum sampling after multiple doses to detect ADAs and to characterize their specificity (i.e. if they are directed to the antibody or to the payload). If an immunogenic response occurs, changes to the construct design (peptide sequences or linkers) or immune tolerance induction strategies will be required.

Studies on other biologic therapies for AD, such as mAbs targeting Aβ, have shown that immunogenicity can be a challenge, with some patients developing ADAs that affect treatment efficacy [126, 129]. However, the use of humanized or fully human antibodies has reduced this risk. In the case of nanobody conjugates, the smaller size and unique structure of nanobodies may offer advantages in terms of reduced immunogenicity compared to full size antibody [125, 126]. Additionally, the selection of payloads with low immunogenic potential, such as small molecules or peptides with known safety profile can further minimize the risk [76, 125]. Clinical trials should include monitoring ADAs and assessment of their impact on pharmacokinetics and efficacy.

Ackaert et al. (2021) provided direct evidence that nanobody-based therapeutics tend to have low immunogenicity [129]. The authors examined two nanobodies in clinical development and found that only 1 out of 20 patients had pre-existing ADAs against an anti-HER2 (human epidermal growth factor receptor 2) nanobody, and the ADA amount increased only marginally after 3 months of treatment with no adverse effects on safety or pharmacokinetics [129]. Both tested nanobodies were camelid-derived (non-humanized), yet in ex-vivo assays they showed minimal activation of human dendritic cells and T-cells. Their findings indicate that monomeric VHH domains present a low immunogenicity risk profile, which is very encouraging for therapeutic use [129]. The authors stress that keeping nanobodies monomeric or using careful humanization strategies helps mitigate ADA development [129]. In addition, a review article by Jovčevska & Muyldermans (2020) compares nanobodies to conventional mAbs, especially regarding immunogenicity in the context of therapy [130]. The authors note that nanobodies have been observed in multiple studies to induce little or no anti-drug antibody response in vivo [125, 130]. One reason is that the VHH nanobodies can be humanized by grafting onto human heavy-chain frameworks, greatly reducing T-cell epitopes [125]. Also, a single-domain nanobody presents a smaller surface for the immune system to recognize, compared to a full IgG (~ 150 kDa) with foreign constant regions [125]. The authors cite clinical experiences (e.g., with Caplacizumab) where patients rarely developed neutralizing antibodies, and even when low-titer ADAs occurred, they did not compromise efficacy [130]. For instance, the first approved antibody drug Caplacizumab showed a low incidence of treatment-emergent ADAs (~ 3.9% of patients across trials) with no impact on clinical outcomes [130]. Thus, the authors highlight that nanobodies, especially if properly humanized, are far less immunogenic than earlier-generation biologics, making them attractive for chronic use in diseases such as AD [130].

## Preclinical and clinical translation path

Rigorous testing in multiple AD models during the transition from pre-clinical research to clinical trials is required to confirm efficacy and safety. First, familial AD transgenic mouse models are used, with possible extension to aged primates if they demonstrate amyloid pathology. Alternatively, human stem cell-derived brain organoids offer a complementary system to model amyloid accumulation in a more human genetic background, however, the organoid model requires careful development to ensure it accurately reflects disease-relevant features. These models will determine the therapy’s impact on neuroinflammation, amyloid clearance, and neuronal function, and the data will be critical for clinical translation [131] (Table 4).

**Table 4:** A framework of translational study

Component	Details	Purpose
Model system	Brain organoid, APP mice, tau transgenic mice, aged primates	Assess efficacy on amyloid, tau, and neuroinflammation
Biomarkers	PET([^18^F]florbetapir), MRI, CSF (Aβ42, tau, IL-1β, TNF-α),	Monitor target engagement and therapeutic response
Trial structure	Phase 1 dose-escalation in mild AD patients	Evaluate safety, pharmacokinetics, preliminary efficacy

A major strength is the use of anti-Aβ amyloid fibril antibodies that are already approved or under clinical investigation. This reduces the safety risks concerned related to the antibody component. However, incorporating a payload requires GLP (Good Laboratory Practice) toxicology studies in at least two species, including neurobehavioral assessments, to determine the highest safe dose for the first-in-human study [131, 132]. The route of administration must be determined based on preclinical findings. If BBB permeability is insufficient, a Phase 1 trial may use intrathecal delivery to confirm brain uptake and validate the therapeutic approach. If BBB penetration strategies, such as RMT, prove highly effective, IV administration may be considered. However, magnetic resonance imaging (MRI) monitoring is necessary to detect potential adverse effects, including ARIA or inflammation. Additionally, CSF cytokine profiles, such as decreased IL-1β or increased IL-10, may indicate successful immune modulation [62, 124, 128].

Recent preclinical studies have begun to demonstrate the feasibility and efficacy of nanobody-based conjugates in AD models, though each comes with important limitations (Table 5). For example, Zhao et al. engineered a multivalent nanobody conjugate by grafting Aβ-binding peptide and an interleukin-1β (IL-1β) fragment into a single nanobody, then linking it to a rigid ROS-scavenging polymer scaffold [133]. In AD transgenic mice, this multivalent conjugate significantly attenuated memory deficits and pathology by inhibiting amyloidogenesis, reducing oxidative stress, and modulating neuroinflammation [133]. This study provides proof of concept for the targeted delivery of anti-inflammatory payloads and antioxidant payloads via nanobodies to sites of amyloid pathology, supporting the therapeutic strategy proposed in this review. However, the experiment was limited to small cohorts of mice with relatively short-term observation, and only a single dosage regimen was reported, meaning that dose–response efficacy and long-term safety remain uncharacterized. While the therapeutic effects were clear in this isolated study, broader validation is needed in additional models and with larger samples to ensure reproducibility.

**Table 5:** Comparative summary of in vivo therapeutic nanobody studies in Alzheimer’s disease models

Study (year)	Nanobody/ Target	Therapeutic payload	AD model	Administ- ration route	Key outcomes	Limitations
Zhao et al., 2023 [133]	Multivalent nanobody (Aβ)	ROS scavenger scaffold + IL-1β fragment	AD transgenic mice	Intravenous	Reduced amyloid and oxidative stress; improved cognition	Small cohort; single dose; short follow-up (weeks)’ no dose–response analysis
Marino et al., 2022 [145]	Anti-BACE1 nanobody	AAV-mediated gene therapy	AppNL-G-F knock-in mice	Intravenous (single AAV-dose) – amyloid model	Long-term (> 12 months) Aβ and neuro-inflammation reduction; cognitive benefits	Moderate cohort size; single-dose; single-cohort study; conducted in one mouse model; limited dose exploration
Kang et al. 2022 [146]	BBB-shuttle VHH + Aβ- binding peptide (KG207-M)	Fusion construct	McGill Thy1-APP rats	Intravenous (weekly dosing)	Reduced brain Aβ, improved biomarkers and MRI measures	Small cohort; single dosing regimen; intermediate follow-up (5 weeks); no dose–response
Danis et al. 2022 [147]	Intracellular anti-tau nanobody (Z70)	AAV-mediated intrabody	Tau seeding mouse model (P301S)	Intracranial/AAV	Reduced tau pathology propagation	Small cohort; single-dose gene therapy; short follow-up (weeks-months); no behavioral outcomes
Haynes et al. 2024 [134]	Alpaca-derived nanobody (E3)	Fluorescein (diagnostic probe)	5XFAD mice	Intravenous	Crossed BBB, bound soluble Aβ oligomers and plaques	Very small cohort; single imaging dose; short-term imagine study (acute post-injection)
Vandesquille et al. 2017 [135]	Anti- Aβ nanobody (R3VQ)	Gadoliniums chelate (MRI contrast agent)	*APP* transgenic mice	Intravenous	BBB crossing, selective plaque labeling, enhanced MRI signal	Small cohort; single-dose administration; short-term imaging follow-up (days)
Li et al. 2016 [136]	Anti- Aβ and anti-tau nanobody	Imaging probes	APP/PS1 and tau mice	Intravenous	Real-time imaging of plaques and tau tangles in vivo	Small cohort; single-dose imaging; short-term observation

Furthermore, other studies have shown that nanobodies can be engineered to cross the BBB and even target specific brain regions, enhancing their therapeutic potential [76]. For instance, an alpaca-derived nanobody (clone E3) was developed to target soluble Aβ oligomers, the neurotoxic precursors to plaques [134]. In a 5×FAD AD mouse model, fluorescein-labeled E3 nanobody was able to cross the BBB and bind in vivo to both soluble Aβ oligomers and insoluble amyloid plaques, as observed in the brains of treated mice. Imaging revealed that nanobody-tagged Aβ oligomers clustered around neurons, while plaques were found in the extracellular space, highlighting their distinct morphology and spatial distribution [134]. These findings validate the E3 nanobody as a brain-penetrant diagnostic probe with potential theragnostic (combined therapy and imaging) applications in AD [134]. Yet, this too was a single-study demonstration focused on imaging rather than therapy, no therapeutic intervention or cognitive benefit was assessed. The sample size was modest, and the observation period short (brains were examined acutely after nanobody administration). Therefore, the sustainability of brain targeting and any functional impact remain unknown.

In a related line of research, Vandesquille et al. (2017) engineered an MRI contrast agent by site-specific conjugation of a gadolinium chelate to a nanobody (VHH "R3VQ") that binds Aβ deposits [135]. After IV injection in APP transgenic mice, this gadolinium–nanobody probe efficiently crossed the BBB and labeled amyloid plaques, resulting in greatly enhanced MRI signal and enabling detection of Aβ plaques both in vivo and in postmortem AD mouse brain tissue [135]. This study offered proof-of-concept that nanobodies can be tailored as precise, BBB-permeable imaging agents for amyloid pathology [135]. On the other hand, the experiment involved only a handful of mice (*n* = 3–5 per group) and used a single dose (50 mg/kg) [135]. Plaque labeling was confirmed shortly after administration [135], but long-term retention or repeated imaging was not explored. As a diagnostic tool, this is promising, but from a therapeutic standpoint it remains an indirect illustration, demonstrating distribution and target engagement but not disease modification.

Building on these advances, Li et al. (2016) introduced two novel camelid nanobodies, one targeting extracellular Aβ plaques and another targeting intracellular tau tangles, and demonstrated their use in real-time in vivo brain imaging [136]. Upon IV administration in AD transgenic mice, both nanobodies gradually crossed the BBB and diffused into brain tissue, as visualized by two-photon microscopy. The anti-Aβ nanobody selectively bound to amyloid plaques, while the anti-tau nanobody labeled neurofibrillary tangles within neurons, effectively "lighting up" hallmark AD lesions in the living brain [136]. This demonstrates the exquisite specificity of nanobodies in a complex in vivo environment. As with the other imaging studies, the scope was limited to showing target binding and visualization. The therapeutic impact was not assessed, and the work was confined to a short imaging window in a small number of animals. No dose-ranging studies were reported to determine the minimum effective concentration for clear imaging, which would be analogous to dosing considerations for therapy.

Collectively, these studies demonstrate in principle that nanobody-based conjugates can reach amyloid plaques and other AD pathology in vivo, crossing the BBB and exerting biological effects or imaging contrast. This directly addresses the concern that no conjugate had ever reached plaques in living brains; the examples above show the approach is feasible. At the same time, the evidence so far leans heavily on single-study anecdotes. Each report is essentially a one-off proof of concept with its own engineered construction and unique mouse model, lacking replication or longitudinal follow-up. Several critical qualifiers must be acknowledged when evaluating the current body of research on nanobody studies for AD. Firstly, the studies were constrained by small sample sizes, typically involving only a few mice per treatment group. Secondly, the follow-up periods were relatively short, spanning days to weeks rather than the months or years necessary to capture long-term effects relevant to a chronic condition like AD. Thirdly, the research lacked comprehensive dose–response analyses and long-term toxicity assessments, leaving key questions about optimal dosing and safety unanswered. These constraints suggest that while the feasibility of nanobody conjugates has been established, their therapeutic potential and safety profile remain further to be explored.

To provide a comprehensive evaluation, we have synthesized key in vivo nanobody studies into a structured comparative format (Table 5), detailing their experimental designs, outcomes, and identified limitations. This table encompasses critical aspects of each study, including nanobody targets, therapeutic payloads, animal models, dosing regimens, observed outcomes, and methodological constraints. This analytical framework underscores significant gaps in the current evidence base, such as limited cohort sizes, the absence of rigorous dose–response analyses, and short follow-up periods. Addressing these limitations through larger cohorts, detailed dose–response studies, and extended longitudinal assessments will be essential for robustly validating and advancing nanobody-based therapeutic strategies for AD.

For early translational studies of nanobody-drug conjugates, appropriate biomarkers must be selected to monitor therapy engagement and response. PET imaging with amyloid tracers can assess cerebral amyloid burden, while MRI can evaluate neurodegeneration and structural brain changes over time. In addition, CSF biomarkers, such as Aβ40, Aβ42, total tau, phosphorylated tau, and inflammatory markers (e.g., IL-1β, TNF-α), can provide molecular readouts of disease progression and therapeutic effects. We envision a first-in-human trial beginning with a Phase 1 dose-escalation study in patients with mild AD, primarily assessing safety and pharmacokinetics of the nanobody conjugate, but also exploring preliminary efficacy signals. Cognitive assessments (e.g., ADAS-Cog, MMSE) and biomarker measurements would be incorporated to detect any early trends in clinical or biochemical improvement [133, 137, 138]. Imaging endpoints could be included as well; for instance, amyloid PET to confirm reduction or stabilization of plaque load, and MRI to monitor changes in hippocampal volume or inflammation. Such a trial design aligns with recent guidelines for AD therapeutic testing, emphasizing combined cognitive and biomarker outcomes in early-phase studies [133].

Transitioning nanobody conjugates from bench to bedside will also require overcoming several manufacturing and formulation challenges. Specific numerical details, such as target human dose or cost of goods estimates, are typically determined in the later stages of drug development and are not included here, as the review focuses on the conceptual framework and feasibility of nanobody mediated therapy.

Nanobodies, as recombinant proteins, can be produced using standard biotechnological methods, ensuring scalability and consistency [139–141]. Indeed, their small size and single-domain structure often confer highly yields and stability in production, as demonstrated by prior biopharmaceutical developments [135]. However, the bioconjugation process, attaching therapeutic payloads or scaffolds to the nanobody, must be optimized to produce a homogeneous product with defined stoichiometry. Advanced bioconjugation techniques, such as click chemistry or enzyme-mediated ligation, allow site-specific attachment of payloads, minimizing batch-to-batch variability and preserving the nanobody’s binding affinity [135, 142, 143]. Additionally, rigorous formulation studies are necessary to ensure that the final product remains stable (e.g., no aggregation or loss of activity) during storage and delivery. Nanobody conjugates may require specialized buffers or lyophilized formulations to maintain stability over time. Despite these hurdles, the modular design of nanobody conjugates allows for flexibility and optimization, supporting the transition from bench to bedside [139]. The antibody fragment, linker, and payload can each be fine-tuned or swapped to improve properties without starting from scratch. With continued refinement in production and conjugation techniques, the path to clinical-grade nanobody therapeutics is increasingly feasible. Encouragingly, several nanobody-based agents for other diseases have already reached clinical trials, highlighting that the scale-up and regulatory production of nanobodies is an achievable goal [130, 135, 144].

## Considerations for systematic preclinical synthesis

To further provide considerations for preclinical synthesis, we summarize dosing data from published studies on nanobody-mediated therapies for AD. For instance, Haynes et al. (2024) developed a nanobody (E3) targeting soluble Aβ oligomers in 5 × FAD mice. They administered a single IV dose of 1.8 mg/mL (150 μL, approximately 9–13.5 mg/kg assuming 20–30 g mice) and confirmed detection of Aβ deposits at 4 and 24 h post-injection, with cohort sizes of *n* = 3–5 per group [134]. Although this study focused on imaging rather than therapeutic efficacy, it demonstrated the ability of nanobodies to cross the BBB and bind to amyloid fibrils in vivo. Zhao et al. (2023) reported a multivalent nanobody conjugate (PNBIL) targeting Aβ aggregation and oxidative stress in APP/PS1 mice, achieving significant reductions in Aβ burden (~ 40% by immunofluorescence), ROS, and neuroinflammation, alongside improved cognitive performance after a single dose (dose not specified, *n* = 6–8). However, the study lacked dose-ranging data and long-term follow-up [133]. Abskharon et al. (2023) evaluated a bispecific nanobody targeting tau fibrils in wild-type mice, achieving brain concentrations of 4.3–8.1 µg/g at 30 min post-dose (20 mg/kg IV, *n* = 3), indicating robust BBB penetration but no therapeutic endpoints [148]. Vandesquille et al. (2017) used a nanobody (VHH "R3VQ") conjugated with a gadolinium chelate for MRI imaging of Aβ plaques in PS2APP transgenic mice, administering 50 mg/kg IV (*n* = 3–5), which enhanced MRI signal at plaques within hours but lacked long-term data [135]. Li et al. (2016) employed nanobodies for real-time imaging of Aβ plaques and tau tangles in PS2APP mice (10–50 mg/kg IV), confirming colocalization with plaques via two-photon microscopy, but focused solely on imaging [136].

Regarding chemical linkers that connect payloads to nanobody, existing work adopts both non-cleavable and cleavable types. For example, Haynes et al. (2024) used a stable thioether linker for fluorescein conjugation, ensuring > 95% stability in plasma over 24 h, which is suitable for imaging but not for therapeutic payload release [134]. Zhao et al. (2023) employed a detachable linker (not specified, likely enzyme-sensitive) to release an IL-1β fragment and ROS scavenger, although the linker stability metrics were not reported [133]. Across these studies, cohort sizes averaged *n* = 3–6, follow-up periods ranged from hours to weeks (median ~ 1 week), and efficacy outcomes (where applicable) showed 30%–40% pathology reduction. These findings underscore the feasibility of nanobody-based approaches but highlight limitations, including small cohorts, lack of dose–response data, and short follow-up periods. Future studies should incorporate larger cohorts (*n* > 20), multi-dose escalation (e.g., 5–50 mg/kg), extended follow-up (> 6 months), and detailed linker stability metrics (e.g., > 90% intact in plasma for cleavable linkers, > 95% for non-cleavable linkers) to bridge toward clinical translation.

## Quantitative considerations for translational planning

To provide references for translational planning, we extrapolate quantitative metrics from approved ADCs and nanobody therapeutics. Chemistry, manufacturing, and controls (CMC) timelines for ADCs average 24–30 months for traditional development to Phase 1 Investigational New Drug (IND) filing, but can be accelerated to 15 months or less with integrated strategies such as one-stop CRDMO platforms, as demonstrated in multiple ADC programs including those supported by WuXi XDC. ADC manufacturing yields for the antibody component typically range 0.5–2 g/L in mammalian cells, as observed in approved ADCs like sacituzumab govitecan, while conjugation efficiencies aim for drug-to-antibody ratios of 7–8. For nanobody therapeutics, CMC timelines are often shorter due to simpler microbial production, averaging 9–18 months to IND filing, with production yields of 60–200 mg/L in *E. coli* systems, enabling cost-effective scale-up for clinical supply [149].

Cost estimates for CMC development to Phase 1 IND filing for ADCs typically range from $3–6 million for non-clinical activities, with approximately $3.1 million allocated to CMC efforts alone, driven by the complexity of antibody production, linker-drug synthesis, and bioconjugation processes. Manufacturing costs per gram for ADCs average $500–800 in standard facilities, incorporating raw material expenses such as $50–300/g for the mAb component and $4000/g for drug-linkers, although process-intensified methods like solid-phase conjugation can reduce this to approximately $85/g with optimized column dimensions and yields. In contrast, nanobody therapeutics exhibit substantially lower production costs of $3–4/g when expressed in fermenter-based systems like *Pichia pastoris*, or generally below $100/g using prokaryotic hosts such as *E. coli* or yeast, facilitating more affordable CMC development—potentially 50% lower than ADCs—due to simpler expression, purification, and scalability.

## Conclusions and future directions

The advent of nanobody-mediated neuroinflammatory therapy heralds a paradigm shift in the treatment of AD, moving beyond the limitations of amyloid-centric approaches to address the pervasive role of chronic neuroinflammation. This strategy delivers anti-inflammatory payloads to Aβ plaques with the precision of sdAbs and thereby attacks the inflammatory epicenters of AD,activated microglia and astrocytes,without the systemic side effects of conventional anti-inflammatory drugs or the immune-related side effects of traditional mAbs. With their compact structure and lack of Fc regions, sdAbs have better tissue penetration and a lower likelihood of causing adverse inflammatory responses and thus hold promise for the development of next-generation therapeutics.

This approach not only fills a critical gap in current AD treatment paradigms but also sets the stage for synergistic interventions. Combining plaque-specific immunosuppression with amyloid-clearing therapies could amplify therapeutic outcomes, halting both the structural and inflammatory cascades of AD progression. The adaptability of the sdAb platform further spurs innovation, allowing customized payloads and delivery systems to match the disease’s complexity as new insights of AD pathogenesis emerged.

Further rigorous investigation is required before this approach can be implemented in clinical practice. Key areas for future research include mechanistic studies to clarify how the payload interacts with glial cells and to improve therapeutic specificity, as well as longitudinal assessments to evaluate long-term efficacy and safety in preclinical models. Optimizing delivery systems, such as non-invasive intranasal administration, may enhance brain uptake and patient compliance. Immunogenicity studies are necessary to confirm that sdAbs have low immunogenic potential with repeated administration. Combination studies with anti-amyloid or anti-tau therapies may determine whether this strategy provides added benefits. Notably, mAbs and antisense oligonucleotides are also being developed to target tau in AD, complementing nanobody-based approaches [150, 151].

A recent study explored the use of nanobodies for anti-tau treatments [152]. The cytosolic antibody receptor and E3 ubiquitin ligase tripartite motif-containing protein 21 (TRIM21) system can degrade antibody-bound targets through the RING domain [152], but this approach is limited by the inability of antibodies to enter intracellular domains. By fusing the RING domain of TRIM21 to a target specific nanobody (R-Nb), this approach generated genetically encoded degraders that effectively decreased pathological intracellular tau aggregates in both in vitro and in vivo models while preserving normally functioning tau proteins [152]. This study reflects the potential of nanobody treatment beyond anti-amyloid aggregates, showing promise in expanding future studies and applications of nanobody treatments to address the complex pathologies present in AD [152]. The further development of biomarkers and imaging tools will allow real-time assessment of treatment response. Additionally, large-scale production methods and regulatory standards must be established for clinical use. Beyond these approaches, artificial intelligence (AI)-driven methodologies offer additional avenues to enhance the development of nanobody-mediated neuroinflammatory therapies for AD. Machine learning algorithms can facilitate efficient screening and selection of high-affinity, low-immunogenicity sdAbs, significantly accelerating therapeutic candidate discovery. Furthermore, AI-assisted modeling could predict BBB penetration and pharmacokinetic profiles, helping optimize targeted delivery and therapeutic efficacy [153, 154]. Integrating AI analyses with emerging biomarker and imaging tools could also enhance personalized intervention strategies, thereby streamlining clinical development pathways. Thus, the inclusion of AI represents a transformative opportunity to advance precision medicine in AD therapeutics. In summary, recent advancements in these areas could reshape AD treatment by preserving cognitive function and slowing disease progression. Nanobody-mediated neuroinflammatory therapy represents a precise and promising strategy to improve therapeutic efficacy and safety for AD.

## Data Availability

Not applicable.

## Abbreviations

AD: Alzheimer’s disease
APP: Amyloid Precursor Protein
APOE: Apolipoprotein E
BDNF: Brain-derived neurotrophic factor
TSPO: Translocator protein
ARIA: Amyloid-related imaging abnormalities
BBB: Blood–brain barrier
ROS: Reactive oxygen species
TNF-α: Tumor necrosis factor alpha
IL-1: Interleukin
NSAID: Nonsteroidal anti-inflammatory drug
C1q: Complement component 1q
TGF-β1: Transforming growth factor beta 1
ARIA-E: ARIA–edema (vasogenic edema)
ARIA-H: ARIA–hemorrhage (microhemorrhages or superficial siderosis)
CNS: Central nervous system
sdAb: Single-domain antibody
NLRP3: NOD-like receptor family pyrin domain-containing 3
MAPK: Mitogen-activated protein kinase
NP: Nanoparticle
LNP: Lipid nanoparticle
CSF: Cerebrospinal fluid
IV: Intravenous
RMT: Receptor-mediated transcytosis
ADA: Anti-drug antibody
PET: Positron emission tomography
MRI: Magnetic resonance imaging
ADC: Antibody–drug conjugate
AI: Artificial intelligence
TfR: Transferrin receptor

## Declarations

### Disclaimer

This research was supported [in part] by the Intramural Research Program of the National Institutes of Health (NIH). The contributions of the NIH author(s) are considered Works of the United States Government. The findings and conclusions presented in this paper are those of the author(s) and do not necessarily reflect the views of the NIH or the U.S. Department of Health and Human Services.
